# Effect of Different Working Settings of Sandblasting on Resin Composite Repair Bond Strength

**DOI:** 10.3390/ma18020313

**Published:** 2025-01-12

**Authors:** Clemens Lechte, Erik Hohmann, Annette Wiegand, Philipp Kanzow

**Affiliations:** 1 Department of Preventive Dentistry, Periodontology and Cariology, University Medical Center Göttingen, 37075 Göttingen, Germany; clemens.lechte@med.uni-goettingen.de (C.L.); annette.wiegand@med.uni-goettingen.de (A.W.); 2 Department of Restorative Dentistry, Periodontology and Endodontology, University Medicine Greifswald, 17475 Greifswald, Germany

**Keywords:** aluminum oxide sandblasting, composite repair, restoration repair, shear bond strength, SBS, working setting

## Abstract

To investigate the effect of different sandblasting settings on the shear bond strength (SBS) in the repair of resin composite, specimens (resin composite, enamel, and dentin; each group *n* = 16) were sandblasted by varying the parameters of air pressure (0.2/0.3/0.4 MPa), angle (45/90°), particle size (27/50 μm), tip size (0.8/1.2 mm), and distance (2/5/10 mm) prior to the application of a universal adhesive (Adhese Universal) and resin composite (adhesive area: 7.07 mm^2^). The specimens were subjected to artificial aging (10,000 cycles, 5–55 °C) prior to (resin composite only) and after repair. Groups without mechanical pretreatment and resin composite incremental bond strength served as controls. Statistical analysis was performed using ANOVAs, post hoc tests, and Chi^2^-tests (*p* < 0.05). Only air pressure and distance impacted SBS (*p* ≤ 0.049). However, resin composite SBS did not differ from the resin composite incremental SBS within all sandblasting settings (positive control: 21.0 ± 5.0 MPa, *p* ≥ 0.566). While sandblasting did not impact bond strength on enamel (control: 20.5 ± 5.1 MPa, *p* ≥ 0.999), most settings resulted in a lower bond strength on dentin (control: 20.1 ± 4.7 MPa, *p* ≤ 0.027). In conclusion, sandblasting significantly improves resin composite repair bond strength, while application parameters are of minor relevance.

## 1. Introduction

Resin composite restorations most often fail due to secondary caries, fractures, and aesthetic compromise [1]. Instead of complete replacement, partially defective restorations are frequently and successfully repaired using resin composite [2,3,4,5].

Repairs are suitable for the minimally invasive management of partially defective restorations. As opposed to complete replacement, more tooth substance is preserved, potentially harmful effects to the pulp and iatrogenic damage to adjacent teeth are reduced, and the treatment time and costs are often reduced. Clinical situations suitable for repairs might include marginal problems (e.g., secondary caries), surface problems, or fractures and the partial loss of the restoration or adjacent tooth substance (e.g., chipping, cusp fractures, and endodontic access cavities). However, repairs are not feasible/possible if the remaining restoration is insufficient/loose, if multiple/generalized problems occur, or if the defect is not accessible (e.g., deep undermining caries) [6].

Creating a sufficient bond between the existing restoration and the repair resin composite is crucial. The repair surface is usually treated with different physical and/or chemical conditioning measures to create a microretentive surface [7]. In addition to the use of buffered hydrofluoric acid on silicate ceramics, airborne particle abrasion can be performed on all restorative materials [8].

For the intraoral sandblasting of partially defective restorations, different repair systems are commercially available (e.g., CoJet Prep, 3M Espe, St Paul, MN, USA; MicroEtcher CD, Danville Materials, Carlsbad, CA, USA). Either aluminum oxide (varying particle sizes are available) or 30 µm silica-coated aluminum oxide (e.g., CoJet sand, SilJet powder) are used as blasting agents. In addition to creating a microretentive surface, silica-coated particles allow for the tribochemical coating of the existing restoration and chemical bonding to subsequently applied silane coupling agents.

Detailed instructions on the use of sandblasting and the exact parameters (i.e., blasting agent, particle size, air pressure, working time, distance, and angle) to be used differ widely between different protocols for restoration repair. A recent systematic review of repair protocols identified the recommended air pressure for resin composite repairs to vary between 0.23 and 0.48 MPa and application times between 4 and 15 s [9]. According to the manufacturers’ instructions for use, the CoJet system is designed for both aluminum oxide (40–60 µm, 0.2–0.7 MPa) and silica-coated aluminum oxide (recommended pressure: 0.2–0.3 MPa). The MicroEtcher CD is also suitable for aluminum oxide (50–90 µm, 0.23–0.7 MPa, recommended pressure: > 0.4 MPa) and silica-coated aluminum oxide. Both manufacturers advise holding the tip 2–10 mm above the restoration surface. However, dentists cannot directly control some of these parameters (e.g., air pressure) using the above-mentioned devices, and the clinical situation also limits working settings (e.g., working angle).

Regarding the impact of sandblasting under different settings, only one study investigated the effect of different working distances on resin composite repair bond strength, but it found no differences in repair bond strength [10]. However, a previous study showed that varying working settings (particle size, air pressure, working time, and working distance) impacted surface roughness, and both varying particle size and air pressure resulted in different metal-ceramic bond strength [11].

Due to the above-described heterogeneity in recommended working settings, the present study aimed to jointly assess the effect of various working settings on resin composite-composite repair bond strength, as well as the bond strength on the adjacent tooth structure. The null hypothesis tested was that the repair bond strength and potential side-effects on the adjacent tooth substance are not affected by different sandblasting working settings.

## 2. Materials and Methods

### 2.1. Preparation of Specimens

All specimens were prepared by a single operator (E.H.). Further details and application parameters of the materials used throughout the study are provided in Table 1.

Cylindrical resin composite specimens were fabricated by placing 2 mm thick increments (Tetric Prime A2, Ivoclar, Schaan, Liechtenstein) into hollow resin cylinders (inner diameter: 6 mm; height: 3 mm; Paladur, Kulzer, Hanau, Germany). Each increment was light-cured according to the manufacturer’s instructions for 10 s (BA Optima 10, BA International, Hamburg, Germany). The light intensity of the curing light (≥1000 mW/cm^2^) was verified after every 20 specimens using a radiometer (Cure Rite Visible Curing Light Meter, Dentsply Caulk, Milford, DE, USA). Enamel and dentin specimens were derived from bovine permanent lower incisors stored in water and taken from slaughterhouses using a diamond-coated trepan drill (custom-made, inner diameter: 5.7 mm; Geb. Brassler, Lemgo, Germany). The specimens were embedded in resin (Paladur, Kulzer, Hanau, Germany). All specimens were polished under water-cooling (Roto-Pol34, Struers, Willich, Germany) using silicium carbide sandpapers of different grit sizes (#800 and #1200, Hermes, Hamburg, Germany; #4000, Messner, Oststeinbek, Germany). Subsequently, specimens with any macroscopically visible irregularities were excluded from the subsequent experiments (<3%), and resin composite specimens were subjected to artificial aging (10,000 cycles, 5–55 °C) using a thermocycler (Willytec Thermocycler V2.9, Gbr. Haake, Karlsruhe, Germany).

### 2.2. Mechanical and Chemical Pretreatment

**Figure 1:**
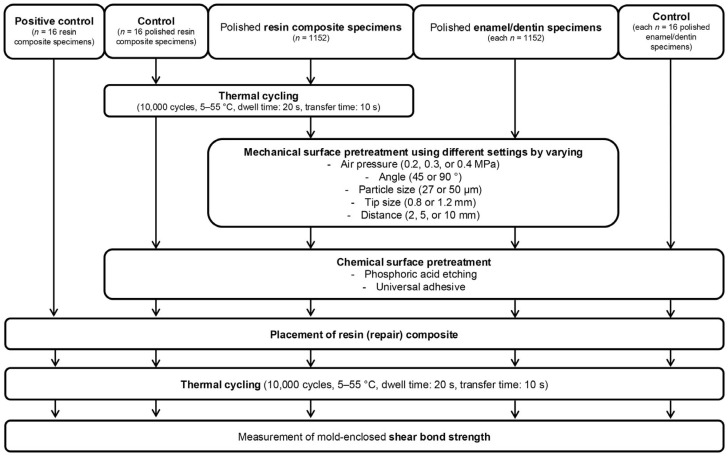
Study design.

Subsequently, specimens were randomly subjected to different mechanical surface pretreatments to assess the effect of different working settings on the (repair) bond strengths (Figure 1): air pressure (0.2, 0.3, or 0.4 MPa), angle (45 or 90°), particle size (27 or 50 µm), tip size (0.8 or 1.2 mm), and distance (2, 5, or 10 mm). This resulted in a total of 72 experimental groups, each representing a different combination of working settings (each *n* = 16). Sandblasting was performed for about 4 s until the whole surface of the specimen was evenly roughened. A custom-made holding device was used to exactly vary the distance and angle of the sandblaster (MicroEtcher CD, Danville Materials, Carlsbad, CA, USA). Air pressure was adjusted and verified using a pressure regulator (ARP20-F01E, SMC, Tokyo, Japan).

After the mechanical surface pretreatment, enamel and dentin specimens were acid-etched using 35% phosphoric acid (Ultra-Etch, Ultradent Products, Cologne, Germany) for 30 or 15 s, respectively. Phosphoric acid was also applied on resin composite surfaces (15 s) to simulate a contamination which usually occurs during etching of the adjacent tooth structure in the clinical setting. Afterwards, the specimens were thoroughly rinsed with water and air-blown. A universal adhesive (Adhese Universal, Ivoclar Vivadent, Schaan, Liechtenstein) was applied according to the manufacturer’s instructions and polymerized for 10 s (BA Optima 10, BA International, Hamburg, Germany).

Additionally, resin composite, enamel, and dentin specimens without any mechanical surface pretreatment served as the control (each *n* = 16). Within control groups, only chemical surface pretreatment (i.e., phosphoric acid etching and the application of a universal adhesive) was performed.

### 2.3. Simulated Repair

The (repair) resin composite was placed in transparent polyacrylic cylinders (inner diameter: 3 mm) that were fixed on top of the specimens using a custom-made device. Again, resin composite (Tetric Prime A2, Ivoclar Vivadent, Schaan, Liechtenstein) was applied in 2 mm thick increments and light-polymerized according to the manufacturer’s instructions for 10 s (BA Optima 10, BA International, Hamburg, Germany). The light intensity of the curing light (≥1000 mW/cm^2^) was verified after every 20 specimens (Cure Rite Visible Curing Light Meter, Dentsply Caulk, Milford, DE, USA). Afterwards, the polyacrylic cylinders were not removed.

The resin composite incremental adhesion strength served as a positive control: the surface of the resin composite specimens was flattened (CompoRoller, Kerr Hawe, Bioggio, Switzerland) prior to light-curing. Immediately afterwards, the transparent polyacrylic cylinder was applied, and a further increment was placed inside the cylinder.

### 2.4. Shear Bond Strength Testing

Finally, all specimens were (again) subjected to artificial aging (10,000 cycles, 5–55 °C) using a thermocycler, and mold-enclosed shear bond strengths were determined using a universal testing machine (Materialprüfmaschine 1446, Zwick, Ulm, Germany). The maximum force *F* (N) that occurred during the shearing process (1 mm/min parallel to the bonding surface) was recorded by the software (testXpert V12.1, Zwick, Ulm, Germany), and shear bond strength was calculated using the adhesive area *A* (7.07 mm^2^) according to the following equation: *σ = F/A*.

### 2.5. Failure Mode Analysis and Scanning Electron Microscope Imaging

In addition, the fracture sites were analyzed microscopically at ×16 magnification (Stemi SV 11, Carl Zeiss, Oberkochen, Germany) to determine the failure mode. Failure types were classified as adhesive (failures occurring at the bonding interface only), cohesive (failures affecting the base substrate or (repair) resin composite only), or mixed (failures resembling a combination of adhesive and cohesive failures). Also, representative scanning electron microscope (SEM) images of selected experimental groups were taken from the specimens’ surfaces after sandblasting. Specimens were desiccated for 2 weeks in silica gel, sputter-coated with carbon, and analyzed using an SEM at 5 kV (Ultra plus FE-SEM, Zeiss, Oberkochen, Germany).

### 2.6. Statistical Analysis

Statistical analysis was carried out using the software SPPS 29.0 (IBM, Armonk, NY, USA). The level of significance was set to α = 0.05. As a Kolmogorov–Smirnov test indicated the shear bond strength data to be distributed normally, analyses of variance (ANOVAs) were employed. Separately for the three substrates, five-way ANOVAs using the individual parameters as factors (air pressure, angle, particle size, tip size, and distance) were conducted. Subsequently, for insignificant factors, data were pooled, and one-way ANOVAs, followed by Scheffe’s (homogeneous variances) or Tamhane’s (non-homogeneous variances) post hoc tests, were performed. For all specimens with mechanical pretreatment, the distribution of failure modes (adhesive, mixed, or cohesive) was separately assessed for each parameter (air pressure, angle, particle size, tip size, and distance) using Chi^2^-tests. The resulting *p*-values were corrected according to Bonferroni–Holm.

## 3. Results

### 3.1. Shear Bond Strength

For all substrates, only air pressure and distance showed a significant effect on shear bond strengths in the five-way ANOVAs (*p* ≤ 0.049, Table 2). For insignificant factors, data were pooled, resulting in nine different settings (combinations of air pressure and distance parameters). Subsequent one-way ANOVAs were performed for the different substrates (Table 3).

For the resin composite specimens, the shear bond strength of the control (no mechanical surface pretreatment) amounted to 15.2 ± 5.7 MPa. Sandblasting increased the shear bond strength in all settings to values that were not significantly different from the positive control (resin composite incremental bond strength: 21.0 ± 5.0 MPa, *p* ≥ 0.566). On enamel, the shear bond strength was not affected by the different settings when compared to the control (20.5 ± 5.1 MPa, *p* ≥ 0.999). On dentin, most settings resulted in lower shear bond strength compared to the control (20.1 ± 4.7 MPa, *p* ≤ 0.027).

### 3.2. Failure Modes

Regarding the failure mode analysis, the distribution of failure modes (adhesive, mixed, or cohesive) differed between the substrates (*p*_adj_. < 0.001), and resin composite specimens showed a higher proportion of cohesive failures (86.5%) than enamel and dentin specimens (17.5% and 4.8%, respectively). Only for dentin specimens, the settings impacted failure modes (*p*_adj_. < 0.001): larger distances resulted in a higher proportion of cohesive failures (2 mm: 12%, 5 mm: 15%, 10 mm: 28%). Regarding the resin composite specimens, cohesive failure modes dominated (Table 4), but the distribution of failure modes was not different between the settings (*p* ≥ 0.106).

### 3.3. Scanning Electron Microscope Imaging

**Figure 2:**
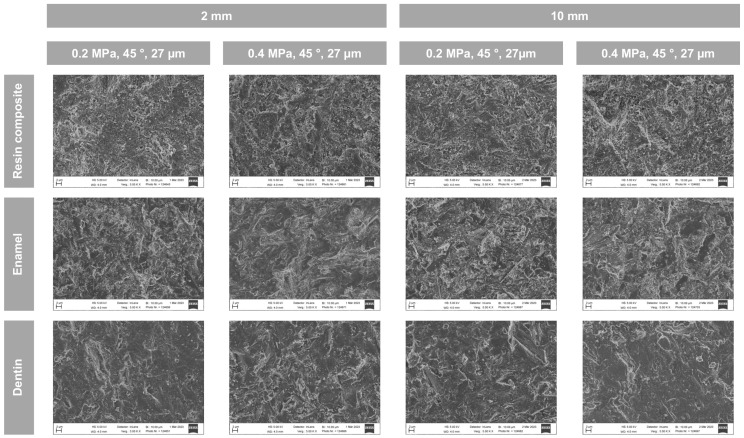
Representative SEM images of selected experimental groups (magnification: ×5000). The large tip size (1.2 mm) was used for all images.

Representative SEM images after sandblasting using exemplary settings (45° angle and 27 µm aluminum oxide) are shown for different air pressures (0.2 vs. 0.4 MPa) and distances (2 vs. 10 mm) in Figure 2. The assessed working settings resulted in a comparable surface topography.

## 4. Discussion

The present study aimed to investigate the effect of different sandblasting settings on resin composite repair bond strength and bond strength on enamel and dentin. Overall, air pressure and distance showed a statistically significant effect on bond strengths. Based on these results, the null hypothesis must be rejected. However, post hoc tests did not reveal any individual superior working setting. All nine working settings with different air pressure and distance (i.e., using pooled data of non-significant factors) resulted in repair bond strengths that did not differ from the resin composite incremental bond strength. These results underline the importance of sandblasting during the clinical procedure to allow for optimal repair bond strength which are statistically not inferior to the resin composite incremental bond strength (positive control).

Representative SEM images showed roughened surfaces after sandblasting in the case of all assessed working settings. Also, a comparable surface topography throughout all working settings was observed. In addition, remnants of abrasive particles were visible, and dentine specimens showed an occlusion of dentinal tubules, presumably due to the smear layer and sandblasting, as images were taken prior to the application of phosphoric acid and the subsequent water-rinsing. This observation is in line with the REM analysis in a previous study showing roughened enamel surfaces and damaged dentin surface, including remnants of abrasive particles and covered dentinal tubules after sandblasting [12].

Within the present study, bovine enamel and dentin were used. Bovine teeth are easily available in large numbers, and they ensure similar properties across all specimens. As shown in a previous systematic review, bovine teeth are comparable to human teeth regarding bond strength tests in adhesive dentistry [13]. So, they seem to be a suitable substrate for testing the influence of sandblasting on bond strengths. Also, a universal adhesive (Adhese Universal) without a silane component was used. The potential effect of chemical pretreatment using a silane (e.g., contained within a universal adhesive with a silane component or a universal primer) was beyond the scope of the present study, which aimed to assess the effect of different sandblasting parameters. Consequently, only aluminum oxide (27 μm, 50 μm) and no silica-coated aluminum oxide were used. The assessed parameters were varied within the ranges frequently mentioned in clinical repair protocols [9] and within manufacturers’ instructions. Despite using universal adhesives, the separate application of phosphoric acid is recommended, at least for enamel surfaces, to achieve optimal bond strength [14]. To simulate potential contamination via phosphoric acid within the clinical setting, phosphoric acid etching of all specimens was performed. A previous in vitro study showed that sandblasting after etching with phosphoric acid destroys the etching pattern on enamel and dentin, resulting in reduced bond strength [12]. Therefore, phosphoric acid etching was applied on all substrates after sandblasting.

Finally, specimens were subjected to artificial aging using thermal cycling. Instead of assessing immediate bond strength, a clinical service time of about 12 months was simulated [15].

To the best of our knowledge, only one previous study assessed the effect of different working settings on the resin composite repair bond strength: Burrer et al. [10] used 50 µm aluminum oxide at 0.3 MPa air pressure for 10 s. The authors found that resin composite repair bond strength does not significantly differ between varying working distances ranging from 1 to 15 mm. This is partially in line with the results of the present study, as post hoc tests did not show any superior working distance.

In addition, previous studies focused on optimal sandblasting parameters to achieve a stable bond between metal and veneering ceramic or between ceramic and luting cement. While these studies do not directly resemble the clinical setting of intraoral repairs, they might add valuable information regarding the surface treatment effect of sandblasting on bond strength. Regarding 50 µm aluminum oxide, Coskun et al. [11] found no significant effect of air pressure (ranging from 0.2 to 0.5 MPa), distance (ranging from 10 to 30 mm), or application time (ranging from 10 to 30 s) on metal–ceramic bond strength. Only when larger particle sizes up to 250 µm were used, air pressure had a significant effect, and higher air pressure resulted in significantly higher bond strength. However, these larger particle sizes are not used for intraoral repair, which was the scope of the present study. Another study by Edelhoff and Marx [16] used 110 µm aluminum oxide and showed that varying air pressure (ranging from 0.025 to 0.4 MPa) and distance (10 vs. 20 mm) are only of minor relevance for the ceramic–composite bond strength, while higher air pressure tended to result in lower bond strength. While these previous studies showed conflicting results regarding the effect of air pressure, higher air pressure settings resulted in slightly, albeit insignificant, lower bond strength in the present study.

As a limitation of the present study, the application time of the sandblasting procedure was not precisely measured. Instead, sandblasting was performed for about 4 s until an even surface appearance was achieved. Notably also, clinical recommendations often contain unprecise information without a specific application time (e.g., five spray applications) [17]. Also, different working times did not impact shear bond strength in a previous study [11].

In the clinical situation, partially defective restorations often require adhesive pretreatment of the adjacent tooth substance [6]. Ideally, all involved substrates should benefit from the applied repair conditioning measures. While intraoral sandblasting is used to improve repair bond strength to the existing (resin composite) restoration, sandblasting might result in reduced bond strength to the adjacent tooth substance. Regarding potential side-effects of sandblasting on the dental hard tissue, previous studies showed significantly reduced bond strength to enamel and dentin if sandblasting was applied after phosphoric acid etching [12,18]. When sandblasting was applied prior to phosphoric acid etching, a previous study showed only a small but not significant reduction in enamel and dentin bond strengths [12]. Consequently, phosphoric acid was applied after sandblasting in the present study. Despite this sequence, a significant reduction in bond strength on dentin (*p* ≤ 0.027) but not on enamel was found in the present study. Therefore, it seems important to cover large areas of adjacent dentin prior to the sandblasting of the resin composite surface to avoid reduced bond strength on dentin [19].

To the best of our knowledge, the present study is the first study to jointly compare the effect of various sandblasting settings prior to resin composite repair. Different parameters often contained in clinical guidelines and manufacturers’ instructions were assessed and the parameters were varied within wide ranges to allow for a comprehensive assessment.

## 5. Conclusions

Sandblasting significantly improves resin composite repair bond strength. In practical use, the application parameters are only of minor relevance. These findings are reassuring, as some of the assessed working settings often cannot be directly controlled by dentists (e.g., air pressure) or working settings may be limited by the clinical situation (e.g., working angle).

## Figures and Tables

**Table 1:** Composition of materials used as described in manufacturers’ safety data sheets and instructions for use.

Product	Manufacturer	Lot No	Composition	Application Mode
Aluminum oxide 27 µm	Danville Materials, Carlsbad, CA, USA	L15CY, L20GZ	Aluminum oxide and other oxides (SiO_2_, TiO_2_)	*Varied throughout the experimental groups*
Aluminum oxide 50 µm	Danville Materials, Carlsbad, CA, USA	L1712, L1V1Q	Aluminum oxide and other oxides (SiO_2_, TiO_2_)	*Varied throughout the experimental groups*
Ultra-Etch	Ultradent Products, Cologne, Germany	BKHNY	Phosphoric acid and dimethicone	Application (resin composite: 15 s, enamel: 30 s, dentin: 15 s), rinsing with water, gentle air-blowing
Adhese Universal VivaPen	Ivoclar Vivadent, Schaan, Liechtenstein	Z01MCG	Methacrylates (HEMA, Bis-GMA, D3MA, methacrylated phosphoric acid ester, DMAEMA), ethanol, water, highly dispersed SiO_2_, initiators (campherquinone), and stabilizers	Application (20 s, scrubbing into the surface using a disposable VivaPen snap-on cannula), gentle air-blowing, 10 s light-curing
Tetric Prime, shade A2	Ivoclar Vivadent, Schaan, Liechtenstein	Z00LBG	Dimethacryltes (UDMA, Bis-EMA, Bis-GMA, D3MA), fillers (barium glass, ytterbium trifluoride, mixed oxide [SiO_2_/ZrO_2_] copolymers), additives, initiators, stabilizers, and pigments	Application (2 mm thick increments), 10 s light-curing

Bis-EMA: bisphenol A diglycidyl methacrylate ethoxylated, Bis-GMA: bisphenol A diglycidyl ether dimethacrylate, D3MA: 1,10-decanediol dimethacrylate, DMAEMA: 2-dimethylaminoethyl methacrylate, HEMA: 2-hydroxyethyl methacrylate, UDMA: diurethane dimethacrylate.

**Table 2:** Results of the five-way analyses of variances (ANOVAs) for each substrate.

Source	Resin Composite		Enamel		Dentin	
	Ratio (F)	*p*-Value	Ratio (F)	*p*-Value	Ratio (F)	*p*-Value
Corrected model (*df* = 8)	4.699	**<0.001**	4.979	**<0.001**	5.103	**<0.001**
Air pressure (*df* = 2)	9.347	**<0.001**	3.020	**0.049**	4.126	**0.016**
Angle (*df* = 1)	0.026	0.872	3.142	0.077	0.054	0.816
Particle size (*df* = 1)	0.158	0.691	3.665	0.056	0.001	0.970
Tip size (*df* = 1)	0.529	0.467	0.922	0.337	0.073	0.787
Distance (*df* = 2)	7.668	**<0.001**	12.955	**<0.001**	9.662	**<0.001**

Significant values are printed in bold. *df*: degree of freedom.

**Table 3:** Bond strengths for different substrates (resin composite, enamel, and dentin) after sandblasting using nine different working settings by varying the parameters’ air pressure and distance (i.e., using pooled data of non-significant factors).

Working Setting		Resin Composite	Enamel	Dentin
0.2 MPa (each *n* = 384)	2 mm	21.0 ± 5.3 ^B^	21.5 ± 4.8 ^ABC^	14.5 ± 5.9 ^AB^
	5 mm	19.3 ± 5.1 ^AB^	21.1 ± 5.4 ^ABC^	15.4 ± 6.2 ^ABCDE^
	10 mm	18.9 ± 4.6 ^AB^	20.1 ± 4.8 ^AB^	15.4 ± 6.0 ^ABCDE^
0.3 MPa (each *n* = 384)	2 mm	21.0 ± 5.2 ^B^	21.8 ± 4.7 ^BC^	13.9 ± 5.0 ^A^
	5 mm	19.6 ± 5.7 ^B^	20.5 ± 4.3 ^AB^	14.8 ± 6.4 ^ABC^
	10 mm	20.7 ± 6.3 ^B^	19.6 ± 5.2 ^A^	14.9 ± 5.5 ^AB^
0.4 MPa (each *n* = 384)	2 mm	19.6 ± 4.3 ^B^	22.2 ± 3.8 ^C^	14.0 ± 4.8 ^A^
	5 mm	18.8 ±4.0 ^AB^	21.3 ± 4.0 ^ABC^	15.9 ± 4.3 ^BE^
	10 mm	18.2 ± 4.2 ^AB^	20.8 ± 4.2 ^ABC^	17.1 ± 4.8 ^CDE^
Control (each *n* = 16)		15.2 ± 5.7 ^A^	20.5 ± 5.1 ^ABC^	20.1 ± 4.7 ^E^
Positive control (each *n* = 16)		21.0 ± 5.0 ^B^	–	–

Mean shear bond strengths ± standard deviations. Within each column, different superscript letters indicate statistically significant differences. Control: specimens without any mechanical surface pretreatment; positive control: resin composite incremental adhesion strength.

**Table 4:** Distribution of resin composite specimens’ failure modes for each parameter.

Parameter		Adhesive		Mixed		Cohesive		Adjusted *p*-Value
		*n*	%	*n*	%	*n*	%	
Air pressure (*n* = 1152)	0.2 MPa	11	2.9	37	9.6	336	87.5	>0.999
	0.3 MPa	10	2.6	42	10.9	332	86.5	
	0.4 MPa	14	3.6	42	10.9	328	85.4	
Angle (*n* = 1152)	45°	13	2.3	49	8.5	514	89.2	0.106
	90°	22	3.8	72	12.5	482	83.7	
Particle size (*n* = 1152)	27 µm	13	2.3	62	10.8	501	87.0	>0.999
	50 µm	22	3.8	59	10.2	495	85.9	
Tip size (*n* = 1152)	0.8 mm	20	3.5	58	10.1	498	86.5	>0.999
	1.2 mm	15	2.6	63	10.9	498	86.5	
Distance (*n* = 1152)	2 mm	10	2.6	34	8.9	340	88.5	>0.999
	5 mm	15	3.9	46	12.0	323	84.1	
	10 mm	10	2.6	41	10.7	333	86.7	
Control (each *n* = 16)		3	18.8	3	18.8	10	62.5	–
Positive control (each *n* = 16)		1	6.3	1	6.3	14	87.5	–

Absolute numbers and row %. Due to the effect of rounding, some numbers do not sum up to 100%. Adhesive: failures occurring at the bonding interface only; cohesive: failures affecting the base substrate or (repair) resin composite only; control: specimens without any mechanical surface pretreatment (i.e., chemical surface pretreatment only); mixed: failures resembling a combination of adhesive and cohesive failures; positive control: resin composite incremental adhesion strength.

## Data Availability

The raw data supporting the conclusions of this article will be made available by the authors upon request.
